# Identification of pain indicators for infants at risk for neurological impairment: A Delphi consensus study

**DOI:** 10.1186/1471-2431-6-1

**Published:** 2006-02-02

**Authors:** Bonnie Stevens, Patrick McGrath, Janet Yamada, Sharyn Gibbins, Joseph Beyene, Lynn Breau, Carol Camfield, Allen Finley, Linda Franck, Alexandra Howlett, Celeste Johnston, Patricia McKeever, Karel O'Brien, Arne Ohlsson

**Affiliations:** 1 Faculty of Nursing, University of Toronto, Toronto, Ontario, Canada; 2 Faculty of Medicine, University of Toronto, Toronto, Ontario, Canada; 3 Research Institute, The Hospital for Sick Children (SickKids), Toronto, Ontario, Canada; 4 Department of Psychology, Dalhousie University, Halifax, Nova Scotia, Canada; 5 Department of Pediatrics, Dalhousie University, Halifax, Nova Scotia, Canada; 6 Interdisciplinary Practice Reseach and Evidence Based Practice, Sunnybrook & Women's College Health Sciences Centre, Toronto, Ontario, Canada; 7 Statistics in Medicine Unit-Research Institute, The Hospital for Sick Children (SickKids), Toronto, Ontario, Canada; 8 Pediatric Pain Service, IWK Health Centre, Halifax, Nova Scotia, Canada; 9 Division of Neurology, IWK Health Centre, Halifax, Nova Scotia, Canada; 10 Department of Anesthesiology, Dalhousie University, Halifax, Nova Scotia, Canada; 11 Centre for Nursing and Allied Health Professions Research, Great Ormond Street Hospital for Children, London, UK; 12 School of Nursing, McGill University, Montreal, Quebec, Canada; 13 Department of Pediatrics, Mount Sinai Hospital, Toronto, Ontario, Canada

## Abstract

**Background:**

A number of infant pain measures have been developed over the past 15 years incorporating behavioural and physiologic indicators; however, no reliable or valid measure exists for infants who are at risk for neurological impairments (NI). The objective of this study was to establish consensus about which behavioural, physiologic and contextual indicators best characterize pain in infants at high, moderate and low levels of risk for NI.

**Methods:**

A 39- item, self-administered electronic survey that included infant physiologic, behavioral and contextual pain indicators was used in a two round Delphi consensus exercise. Fourteen pediatric pain experts were polled individually and anonymously on the importance and usefulness of the pain indicators for the 3 differing levels of risk for NI.

**Results:**

The strength of agreement between expert raters was moderate in Round 1 and fair in Round 2. In general, pain indicators with the highest concordance for all three groups were brow bulge, facial grimace, eye squeeze, and inconsolability. Increased heart rate from baseline in the moderate and severe groups demonstrated high concordance. In the severe risk group, fluctuations in heart rate and reduced oxygen saturation were also highly rated.

**Conclusion:**

These data constitute the first step in contributing to the development and validation of a pain measure for infants at risk for NI. In future research, we will integrate these findings with the opinions of (a) health care providers about the importance and usefulness of infant pain indicators and (b) the pain responses of infants at mild, moderate and high risk for NI.

## Background

Assessment of pain in infants has remained somewhat of an enigma over the past two decades. Although a plethora of infant pain measures has been developed [1], they are infrequently and inconsistently used in clinical practice. This paucity of assessment is particularly evident in infants who are the most vulnerable; such as those at risk for neurological impairment (NI). Infants at varying levels of risk for NI are exposed to multiple painful procedures during their initial days in the NICU [2]. Stevens et al [2], found that during the first day of life, neonates at highest risk for NI experienced the greatest number of painful procedures (e.g., suctioning, heel lances, intravenous starts) compared to lower risk groups and were administered the least amount of opiods [2]. To appropriately manage these at-risk infants, a reliable and valid approach to assessing pain is required.

To address this issue, a comprehensive approach to measurement development taking into account the perspectives of multiple stakeholders such as health care providers from different disciplines, parents and family members needs to be undertaken. Eliciting the perceived importance (in accurately and consistently identifying pain) and clinical usefulness (how feasible and useful the measure is for making decisions concerning pain management) of physiologic, behavioural and contextual indicators that comprise pain measures by experts in infant pain research is an appropriate starting place.

Only a few infant pain measures have taken contextual factors, which consist of any factor either known or thought to influence the infant's pain response (such as gestational age, sleep/wake status, severity of illness, that would assist to describe an infant's pain response within a particular context [3-5]) into consideration. As no measures have incorporated risk for neurological impairment (NI) as a contextual factor, the results of this study will contribute to our understanding as to whether there is a difference in response based on risk for NI or whether this risk in and of itself should be considered a contextual factor. There is inconsistent evidence on the differences in behavioural and physiological indicators when comparing infants with and without NI. Stevens et al [6] reported there were differences in facial activity and heart rate variability with the most at risk for NI infants demonstrating the least response following heel lance. Conversely, Oberlander et al [7] found similar facial and heart rate variability responses to heel lance between groups of preterm infants with and without parenchymal brain injury.

Generally, infants with NI may show fewer and less clear emotional responses [8,9]. For example, infants with Down's syndrome or asphyxia exhibited cries that were less frequent, less variable in intensity and fundamental frequency (pitch) [10-14] and of longer latency from the painful stimulus than cries in infants with no disabilities [10]. Furthermore, there is speculation that differences in facial musculature, hypotonia, and aberrant neural information programming may affect facial pain responses [15,16]. The multidimensional pain response in infants at risk for NI has not been consistently or comprehensively described, and no measure to assess pain in this population has been validated.

Using the Delphi method, the aim of this study was to establish consensus amongst infant pain research experts about which behavioural, physiologic and contextual indicators characterized pain in infants at high, moderate and low risk for NI. The ultimate goal is the development of a new measure or validation of an existing measure for acute pain assessment in this population.

## Methods

### Study participants

A list of 42 local and international infant pain research experts was generated by the study investigators (who represent diverse levels of expertise in medicine [n = 5], nursing [n = 5] and psychology [n = 2]) and from the International Association for the Study of Pain (IASP) Directory of Members, 2004. To support the content validity of the Delphi exercise and to ensure that the most appropriate experts participated in this process, participants were approached if they had at least 2 years of experience in pain research on assessment and management of infants and young children, had published in peer reviewed pain journals, had presented at major pediatric pain meetings or were known to be involved in current research in pediatric pain. Our goal was to include a multidisciplinary (e.g. nurses, physicians, and psychologists), international sample of approximately 10–15 research experts with broad expertise in pain and pain measurement in infants in this Delphi exercise. Although this sample is small, we were drawing from a very finite number of experts with research in a specialized area and thus it is reasonable given the limited number of eligible participants, time commitment required and limitations of international survey research.

### Delphi survey: expert opinions about managing pain in infants at risk for NI

A 39- item self-administered survey was developed by the study investigators to include indicators representing infant physiologic, behavioral and contextual pain responses [2] as well as pain indicators identified by parents and health professionals. This list of indicators was circulated amongst the study investigators (most of whom were infant and pediatric pain experts) to establish face validity. The survey was organized to assess four domains: (a) physical indicators (11 items); (b) facial actions (10 items); (c) vocal behaviour/cry (9 items); (d) infant activity (9 items). The survey was pilot tested for feasibility and content validity with 10 advanced practice nurses from three local neonatal and pediatric intensive care units with expertise in pain assessment and management. The survey was sent electronically to the nurses participating in the pilot study and took approximately 20 to 30 minutes to complete. Based on the pilot study results, minor changes were made to the format of the questionnaire along with the instructions for participants.

Each survey participant was polled individually and anonymously on the comprehensiveness and accuracy of the list of proposed items. Each item was scored on importance and usefulness of the pain indicator for 3 differing levels of risk for NI (i.e., high risk, moderate risk, low risk), which has been defined and verified by a group of neonatal experts for previous studies by this group of researchers[2,17]. The risk cohorts were defined as:

• High risk cohort: i.e., perinatal asphyxia, IVH [Grade III or IV] or a syndrome or chromosomal anomaly;

• Moderate risk cohort: i.e., acute disease processes such as persistent pulmonary hypertension of the newborn, severe meconium aspiration, meningitis, hydrocephalus, necrotising enterocolitis;

• Low risk cohort; i.e., respiratory distress requiring ventilation, sepsis.

Each item on the survey was scored using a 10 point scale, where a score of 1 = not important/useful at all and 10 = extremely important/useful. *Importance *referred to how vital the pain indicator was in accurately and consistently identifying pain following a painful tissue damaging procedure. *Usefulness *referred to the feasibility (i.e., how easily the pain indicator was to observe and score) and clinical utility (i.e., how helpful the pain indicator was for making decisions about individualized pain management)[18]. An indicator could be important for accurately measuring pain but not useful because of the difficulties in practically implementing the indicator to assess pain in clinical practice.

An explanation of the purpose and description of the research was provided to participants with opportunities for clarification of questions prior to completion of the survey. Variable definitions were provided and respondents were asked to add any additional pain indicators they felt were important or useful to the list. Demographic information and level of experience with infants at risk for NI were also obtained from each of participating experts.

All study data were kept confidential by using only a code number on all completed information. Surveys were sent to each potential participant by electronic mail (e-mail) by a research administrative assistant, who was not familiar with the study and who could not link the participant's identity with any response. Any external reporting kept personal information confidential and would only be reported in aggregate form. Completion of the survey was voluntary and return of the completed survey implied consent to participate. This study was part of a larger program of research on pain in infants at risk for NI that was approved by the Research Ethics Boards at the universities and university-affiliated pediatric hospitals participating in the group of studies within this research program.

### The delphi method

The Delphi method, developed by the Rand Corporation in the 1950s, is a research method for eliciting consensus opinions from experts using questionnaires in an iterative process known as rounds [19]. The number of rounds used in the Delphi process varies, although 2–3 rounds are frequently sufficient [20,21]. Questionnaires distributed to experts are both confidential and anonymous [22,23]. Responses from each round are collated and then the same experts are repeatedly requested to complete a revised questionnaire based on the results obtained from previous rounds. Although providing the results from previous rounds may introduce some response bias, the goal of Delphi polling in subsequent rounds is to challenge respondents to determine whether their responses were in agreement with the average responses of the participants. The Delphi is considered complete when there is convergence of opinion or when a point of diminishing returns is reached [22].

The Delphi allows experts to offer their opinions independently and confidentially without the pressures that may occur during face-to-face meetings [22]. Using electronic means, questionnaires can be distributed broadly across a variety of geographical locations and health care disciplines at a lower cost than face-to-face meetings [22]. Participants have the opportunity to alter their opinions in successive rounds [22,24,25] based on the results of previous rounds to achieve consensus. Consensus of items in a survey or questionnaire is indicative of content validity. The Delphi is potentially subject to researcher bias as the researcher controls the extent of opinions requested from the experts and participants are not able to discuss concerns as there is no formal discussion amongst group members where opinions can be challenged or debated [24]. In addition, based on the literature on the Delphi technique, there are no set criteria as to what determines final consensus opinion [22]. Despite these limitations, the Delphi technique has been used successfully as an initial step in instrument development.

### Delphi rounds

We predicted, according to the literature and previous expertise with the Delphi method, that we would require 2 to 3 rounds to reach consensus on pain indicators in infants at risk for NI. After Round 1, the results were tabulated and then reported back to the group. In Round 1, participants were asked to rate the 39 items according to how important and useful each pain indicator was in accurately identifying pain following a tissue-damaging painful procedure in each of the 3 Cohorts of infants. A priori, we decided that a mean rating of 6 or greater on the 10 point scale for importance and usefulness would be maintained as an acceptable level of consensus. All indicators that had a mean rating of less than 6 were dropped from the item list, following Round 1. In Round 2, participants were asked to re-rate the reduced list of items that were based on their aggregated and ranked responses in Round 1. These first two rounds are analogous steps to instrument development where item generation and item reduction would establish the content validity of any future pain measure [26-29]. Participants from Round 1 also participated in Round 2.

### Data management and statistical analysis

All returned surveys were provided with a code number and data were double entered into SPSS version 12 and checked for data entry errors. Descriptive statistics (i.e., means, standard deviations, range) were first conducted prior to the analysis of participant responses in Round 1. Kendall's Coefficient of Concordance was used to determine the agreement of ratings between the expert respondents in Rounds 1 and 2. Mean rankings were based on 39 pain indicators in Round 1 and 21 indicators in Round 2 (Table 1). The sum of the ranks from the pain experts is computed for each of the pain indicators. Kendall's W ranges between 0 (no agreement) and 1 (complete agreement). Statistical significance was assessed using Friedman's non-parametric test. The six observer agreement categories by Landis and Koch [30] were used to describe the strength of agreement between pain experts. A kappa value of 0.00–0.10 is considered poor strength of agreement, 0.11–.20 slight agreement, 0.21–0.40 fair agreement, 0.41–0.60 moderate agreement, 0.61–0.80 substantial agreement and 0.81–1.00 is considered almost perfect agreement [30].

**Table 1:** Pain Variables Used in Delphi Rounds 1 and 2

**Round 1**	**Round 2**
Physical Signs *Decrease in heart rate Increase in heart rate *Decrease respiratory rate *Increase respiratory rate *Decrease blood pressure Increase blood pressure Decrease in oxygen saturation *Increase in oxygen saturation Fluctuations/variations in HR *Change in skin colour *Sweating **Presence of Facial Actions** Facial grimacing (overall) Brow bulge Eye squeeze Naso-labial furrow Open lips *Vertical stretch mouth *Horizontal stretch mouth *Pursed lips Taut tongue *Chin quiver **Vocal Behaviour/Cry** Duration of the first cry Duration of total crying session *Total number of cry bouts *Harshness of cry *High pitched cry *Low pitched cry *Melody of cry-flat *Melody of cry-melodious *Jitters in cry **Activity** Inconsolability Agitation Rigidity of body **Changes in:** Body movements *Head movements *Hand movements *Arm movements *Foot movements Leg movements *items removed in Round 2	**Physical Signs** Decrease in oxygen saturation Increase in heart rate Increase in blood pressure Fluctuations/variations in heart rate **Presence of Facial Actions** Facial grimacing (overall) Brow bulge Eye squeeze Naso-labial furrow Open lips Taut tongue **Vocal Behaviour/Cry** Duration of first cry Duration of the total crying session **Activity** Inconsolability Agitation Rigidity of body **Changes in:** Body movements Leg movements Vocal Behaviour/Cry **Tears **Finger splay **Fighting against ventilator **Lack of or delayed responsiveness ** items added in Round 2

## Results

The demographic information on the participants is described in Table 2. To cover for potential loss to follow-up of respondents through invalid e-mail addresses that were used to invite individuals to participate in the surveys, Round 1 surveys were emailed to 42 infant pain experts identified in July, 2004. Reminder emails were sent to individuals two weeks after the initial email. A total of 6/42 individuals returned an email message stating that they were declining from completing the survey as they felt they were not experts in pain in infants with NI. Twenty-two individuals did not respond to the survey invitation, therefore, we have no information on reasons for nonparticipation. The 28 non respondents consisted of 10 nurses, 8 physicians, 9 psychologists and 1 pharmacist. A total of 14 surveys were returned in Round 1, 5 weeks after the initial survey distribution.

**Table 2:** Demographic Characteristics of Delphi Respondents (N = 14)

Professional role	9 Nurse Scientists
	4 Psychologists
	1 Physical Therapist
Years of experience in current position	13.4 (minimum 5 years; maximum 24 years)
Location of Participant	Canada = 6 United States = 5 Australia = 2 Sweden = 1
Non professional contact with infants who have or at risk for NI	None = 2 Very Little = 8 Moderate amount = 3 Great deal = 1
Professional contact with infants who have or at risk for NI	None = 4 Very Little = 2 Moderate amount = 3 Great deal = 5
Academic or school-based learning about infants at risk for NI	None = 2 Very Little = 3 Moderate amount = 3 Great deal = 6

According to our a priori criteria for item reduction, the list of pain indicators in Round 1 was reduced from 39 to 21. Pain indicators with a mean score of 6 or greater out of 10 were retained and the survey was revised to reflect these changes for Round 2. The revised survey listed each indicator according to mean ratings from the highest to lowest rating within each category. Based on the feedback from experts, 21 new indicators identified in Round 1 were subsumed within an existing indicator or were added as new individual indicators to this survey.

Round 2 surveys were emailed to the 14 respondents from Round 1, 2 weeks after the first Round was completed. A second reminder was sent to respondents and resulted in a return rate of 100% for Round 2.

### Pain indicator ratings for infants at mild risk for NI

The mean rankings for infants at mild risk for NI regarding importance and usefulness of pain indicators are summarized in Table 3. In Round 1, moderate concordance was found across raters (W = .509, p = .001) on the importance of pain indicators. Moderate concordance was also noted across raters (W = .431, p = .001) for usefulness of pain indicators. The top five pain indicators rated from the highest to lowest rankings for importance and usefulness were the same, although the fourth and fifth indicators differed slightly in their ultimate rankings.

**Table 3:** Round One and Two: Top 5 Mean Ranking of Pain Indicators Mild Risk Group

**Rank**	**Important Indicators**	**Mean Rank**	**Important Indicators**	**Mean Rank**
	*Round 1*		*Round 2*	

1	Brow bulge	36.3	Brow bulge	17.5
2	Facial grimace	35.7	Facial grimace	17.0
3	Inconsolability	34.3	Eye squeeze	16.7
4	Agitation	32.8	Inconsolability	15.6
5	Eye squeeze	32.7	Agitation	13.3

**Rank**	**Useful Indicators**	**Mean Rank**	**Useful Indicators**	**Mean Rank**

	*Round 1*		*Round 2*	

1	Brow bulge	35.5	Brow bulge	16.6
2	Facial grimace	34.8	Eye squeeze	16.0
3	Inconsolability	32.0	Facial grimace	15.9
4	Eye squeeze	31.8	Inconsolability	14.3
5	Agitation	31.2	Increased heart rate from baseline	13.8

In Round 2, fair concordance was found across raters (W = .350, p = .001) for importance of pain indicators. Similarly, for mean rankings for usefulness of pain indicators, concordance was fair across raters (W = .270, p = .001). The top five importance pain indicators were the same as the important indicators that were identified in Round 1. The top five usefulness indicators rated in Round 2 were the same as the usefulness indicators identified in Round 1, with the exception of increased heart rate from baseline replacing agitation. Brow bulge, facial expression and eye squeeze were consistently the highest three indicators rated for both usefulness and importance.

### Pain indicator ratings for infants at moderate risk for NI

The mean rankings for infants at moderate risk for NI regarding importance and usefulness of pain indicators are summarized in Table 4. In Round 1, concordance across raters was moderate (W = .447, p = .001) for importance of indicators. The mean rankings for usefulness of pain indicators also showed moderate agreement across raters (W = .416, p = .001). The top five pain indicators rated from the highest to lowest rankings for importance and usefulness were the same.

**Table 4:** Round One and Two: Top 5 Mean Ranking of Pain Indicators Moderate Risk Group

**Rank**	**Important Indicators**	**Mean Rank**	**Important Indicators**	**Mean Rank**
	*Round 1*		*Round 2*	

1	Brow bulge	35.4	Brow bulge	17.5
2	Facial grimace	34.6	Facial grimace	17.1
3	Inconsolability	32.0	Eye squeeze	16.0
4	Eye squeeze	31.7	Inconsolability	15.5
5	Agitation	30.4	Increased heart rate from baseline	13.1

**Rank**	**Useful Indicators**	**Mean Rank**	**Useful Indicators**	**Mean Rank**

	*Round 1*		*Round 2*	

1	Brow bulge	35.4	Brow bulge	16.4
2	Facial grimace	34.6	Facial grimace	15.8
3	Inconsolability	32.7	Eye squeeze	15.0
4	Eye squeeze	32.2	Increased heart rate from baseline	14.6
5	Agitation	30.5	Inconsolability	14.1

In Round 2, mean rankings for importance of pain indicators demonstrated fair concordance across raters (W = .358, p = .001) as did mean rankings for usefulness of pain indicators (W = .268, p = .001). Four out of the five indicators rated for importance were the same as those rated in Round 1, with increased heart rate from baseline replacing agitation. Similarly, the five highest ranking usefulness indicators were the same as those identified in Round 1 with increased heart rate from baseline replacing agitation (as was the case with infants in the mild risk for NI Cohort). Brow bulge, facial expression and eye squeeze were again consistently rated the highest three indicators for both usefulness and importance.

### Pain indicator ratings for infants at severe risk for NI

The mean rankings for infants at severe risk for NI regarding importance of pain indicators are summarized in Table 5. In Round 1, moderate concordance was found across raters (W = .466, p = .001). Mean rankings for usefulness of pain indicators revealed fair concordance across raters (W = .378, p = .001). Four of the five pain indicators with the highest mean rankings for importance and usefulness were the same with the exception of eye squeeze, which was rated important and nasolabial furrow, which was identified as useful.

**Table 5:** Round One and Two: Top 5 Mean Ranking of Pain Indicators Severe Risk Group

**Rank**	**Important Indicators**	**Mean Rank**	**Important Indicators**	**Mean Rank**
	*Round 1*		*Round 2*	

1	Brow bulge	33.8	Inconsolability	17.0
2	Facial grimace	32.6	Facial grimace	16.4
3	Inconsolability	31.6	Brow bulge	16.1
4	Eye squeeze	29.6	Eye squeeze	14.1
5	Agitation	29.5	Fluctuations in heart rate	14.1

**Rank**	**Useful Indicators**	**Mean Rank**	**Useful Indicators**	**Mean Rank**

	*Round 1*		*Round 2*	

1	Inconsolability	33.1	Inconsolability	15.6
2	Brow bulge	31.8	Increase in heart rate from baseline	15.5
3	Facial grimace	30.7	Brow bulge	15.1
4	Agitation	30.6	Facial grimace	14.7
5	Nasolabial furrow	29.5	Reduced oxygen saturation	14.2

In Round 2, mean rankings regarding importance of pain indicators found fair concordance across raters (W = .347, p = .001). Fair concordance was also reported for usefulness of pain indicators (W = .214, p = .002). Four of the five important indicators were the same as those identified in Round 1, where fluctuations in heart rate in Round 2 replaced agitation in Round 1. Increased heart rate from baseline and reduced oxygen saturation in Round 2 replaced agitation and nasolabial furrow in Round 1 for useful pain indicators. Inconsolability was rated the highest important and useful pain indicator for the severe risk group.

## Discussion

This Delphi study resulted in expert consensus and achievement of item generation and reduction in the process of measurement development. In both rounds of the Delphi, consensus was demonstrated among raters as to the most important and useful pain indicators in infants at varying levels of risk for NI. The rankings were relatively similar across groups with the highest ranking indicators that were identified as important and useful for infants in the most severe risk group being inconsolability, facial grimace, brow bulge, eye squeeze, reduced oxygen saturation, increased heart rate from baseline and fluctuations in heart rate. These indicators are similar to the collection of behavioural and physiologic indicators found in some of the most validated measures of infant pain. Facial grimace is included in the Douleur Aiguë du Noveau-né (DAN[31]) a multidimensional behavioural measure, and in two composite pain measures, the Modified Postoperative Comfort Score [32] and the Echelle Douleur Incofort Nouveau-Né (EDIN[33]). The Premature Infant Pain Profile (PIPP) includes brow bulge and eye squeeze, two individual facial actions along with changes in oxygen saturation and heart rate [3,29]. The Modified Postoperative Comfort Score and the EDIN both measure consolability in the infant. Breau et al, [17] reported that health professionals might regard pain as more physiologically based in infants with NI compared to infants with lower risk for NI whose pain responses encompass both physiologic and behavioural indicators. Given the similarity of the findings from this study and existing measures, the question as to whether a new measure is required or whether an existing measure could be revised or expanded needs to be carefully considered.

Strength of agreement between pain experts appeared to be higher in Round 1 compared to Round 2, where most of the experts demonstrated fair agreement when rating pain indicators for infants at varying risks of NI. There is a notable decline in scores in all 3 cohorts between Round 1 and Round 2 for both importance and usefulness as well as the decrease in concordance. The decline in scores is largely attributable to the decrease in the number of indictors from Rounds 1 to 2. The decrease in agreement may reflect the rater's difficulty in making finer rather than broader decisions (e.g. with less items). Conversely, it may reflect the uncertainty that researchers, who have expert knowledge of the importance and usefulness of pain responses but who may not have clinical expertise in directly observing the infants, have in evaluating indicators. Given that the indicators were fairly similar across the mild, moderate, and severe risk groups, one measurement tool might suffice for all infants at risk for NI; however, this dilemma demands that we incorporate the perspectives of both clinical experts and the responses of infants themselves as the "gold standard" in future research to arrive at an ultimate solution to this issue.

## Conclusion

We have used a consensus methodology, the Delphi technique, as the first step in a comprehensive approach to measurement development and/ or validation for assessing pain in infants at risk for NI. The multidisciplinary and international participants were representative of experts in infant pain measurement research. When selected, study participants appeared to have a broad expertise in pain in infants and/ or children with NI; however, in some participants this expertise was less extensive according to participant self-ratings. As the co-investigators on this study had expertise in assessing and managing infants at risk for NI, they were not able to participate in this Delphi consensus exercise. The small number of potential participants reflects the number of available experts in this area of infant pain research. A broader sample of health professionals including physicians and pharmacists might have strengthened the validity of our findings.

Specified inclusion criteria for experts and defining our method for analysis strengthened the Delphi approach. Using electronic methods to allow ease of completing the Delphi exercise (distribution of surveys and reminder emails for completion of surveys) was time- and cost-effective, and provided easy access to the international participants [34]. However, this methodology did not provide us with detailed reasons for nonparticipation. Furthermore, as this study was exploratory in nature and the sample size was small, the results could be influenced by the opinions of outliers. As part of a larger study on pain in infants at risk for NI, parents were concurrently interviewed to obtain their perspectives on how they assessed and managed their infant's pain. Future research will include triangulation of the data from this Delphi consensus exercise with opinions of health care providers and actual infant responses to painful procedures to further develop a reliable and valid pain measure for infants at risk for NI.

## Competing interests

The author(s) declare that they have no competing interests.

## Authors' contributions

All authors contributed to the design of the study, reviewed and approved the final manuscript. JY, JB conducted the statistical analysis. Interpretation of results was primarily conducted by BS, PM, JB, JY, SG.

## Pre-publication history

The pre-publication history for this paper can be accessed here:



## References

[B1] Duhn LJ, Medves JM (2004). A systematic integrative review of infant pain assessment tools. Adv Neonatal Care.

[B2] Stevens B, McGrath P, Gibbins S, Beyene J, Breau L, Camfield C, Finley A, Franck L, Howlett A, McKeever P, O'Brien K, Ohlsson A, Yamada J (2003). Procedural pain in newborns at risk for neurologic impairment. Pain.

[B3] Stevens B, Johnston C, Petryshen P, Taddio A (1996). Premature Infant Pain Profile: development and initial validation. Clin J Pain.

[B4] van Dijk M, de Boer JB, Koot HM, Duivenvoorden HJ, Passchier J, Bouwmeester N, Tibboel D (2001). The association between physiological and behavioral pain measures in 0- to 3-year-old infants after major surgery. J Pain Symptom Manage.

[B5] Hummel PA, Puchalski M, Creech SD, Weiss MG (2003). N-PASS: Neonatal Pain, Agitation, and Sedation Scale- Reliability and validity [abstract]. Pediatric Research.

[B6] Stevens B, McGrath PJ, Gibbins S, Beyene J, Breau L, Camfield C, Finley A, Franck L, Howlett A, Johnston C, O'Brien K, Ohlsson A (2004). Pain responses in neonates at risk for neurologic impairment [abstract]. Journal of Pain.

[B7] Oberlander TF, Grunau RE, Fitzgerald C, Whitfield MF (2002). Does parenchymal brain injury affect biobehavioral pain responses in very low birth weight infants at 32 weeks' postconceptional age?. Pediatrics.

[B8] Fraiberg S, Bullowa M (1979). Blind infants and their mothers: An examination of the system. Before Speech.

[B9] Yoder PJ (1987). Relationship between degree of infant handicap and clarity of infant cues. Am J Ment Defic.

[B10] Fisichelli VR, Haber A, Davis J (1966). Audible characteristics of the cries of normal infants and those with Down's syndrome. Percept Mot Skills.

[B11] Lind J, Vuorenkoski V, Rosberg G, Partanen TJ, Wasz-Hockert O (1970). Spectographic analysis of vocal response to pain stimuli in infants with Down's syndrome. Dev Med Child Neurol.

[B12] Michelsson K (1971). Cry analyses of symptomless low birth weight neonates and of asphyxiated newborn infants. Acta Paediatr Scand Suppl.

[B13] Michelsson K, Sirvio P, Wasz-Hockert O (1977). Pain cry in full-term asphyxiated newborn infants correlated with late findings. Acta Paediatr Scand.

[B14] Michelsson K, Jarvenpaa AL, Rinne A (1983). Sound spectrographic analysis of pain cry in preterm infants. Early Hum Dev.

[B15] Ciccihetti D, Sroufe LA, Lewis M, Rosenblum L (1978). An organizational view of affect: Illustration from the study of Down syndrome infants. The development of affect.

[B16] Ciccihetti D, Beegley M (1990). Children with Down syndrome: A developmental perspective.

[B17] Breau LM, McGrath PJ, Stevens B, Beyene J, Camfield CS, Finley GA, Franck L, Howlett A, O'Brien K, Ohlsson A (2004). Healthcare professionals' perception of pain in infants at risk for neurological impairment. BMC Pediatr.

[B18] Stevens B, Gibbins S (2002). Clinical utility and clinical significance in the assessment and management of pain in vulnerable infants. Clin Perinatol.

[B19] Dalkey NC (1969). The Delphi method: an experimental study of group opinion.

[B20] Procter S, Hunt M (1994). Using the Delphi survey technique to develop a professional definition of nursing for analysing nursing workload. J Adv Nurs.

[B21] Beech B (1997). Studying the future: a Delphi survey of how multi-disciplinary clinical staff view the likely development of two community mental health centres over the course of the next two years. J Adv Nurs.

[B22] Hasson F, Keeney S, McKenna H (2000). Research guidelines for the Delphi survey technique. J Adv Nurs.

[B23] Williams PL, Webb C (1994). The Delphi technique: a methodological discussion. J Adv Nurs.

[B24] Goodman CM (1987). The Delphi technique: a critique. J Adv Nurs.

[B25] Graham B, Regehr G, Wright JG (2003). Delphi as a method to establish consensus for diagnostic criteria. J Clin Epidemiol.

[B26] Lynn MR (1986). Determination and quantification of content validity. Nurs Res.

[B27] McFarland AH, Neale KA, Norman GR, Streiner D (1981). Methodological issues in developing a scale to measure social support. Schizophr Bull.

[B28] Thomas S (1992). Face validity. West J Nurs Res.

[B29] Ballantyne M, Stevens B, McAllister M, Dionne K, Jack A (1999). Validation of the premature infant pain profile in the clinical setting. Clin J Pain.

[B30] Landis JR, Koch GG (1977). The measurement of observer agreement for categorical data. Biometrics.

[B31] Carbajal R, Paupe A, Hoenn E, Lenclen R, Olivier-Martin M (1997). [APN: evaluation behavioral scale of acute pain in newborn infants]. Arch Pediatr.

[B32] Guinsberg R, Kopelman BI, Anand KJ, de Almeida MF, Peres C, Miyoshi MH (1998). Physiological, hormonal and behavioral responses to a simple fentanyl dose in intubated and ventilated preterm neonates. J Pediatr.

[B33] Debillon T, Zupan V, Ravault N, Magny JF, Dehan M (2001). Development and initial validation of the EDIN scale, a new tool for assessing prolonged pain in preterm infants. Arch Dis Child Fetal Neonatal Ed.

[B34] Mandal A, Eaden J, Mayberry MK, Mayberry JF (2000). Questionnaire surveys in medical research. J Eval Clin Pract.

